# Decoding the EEG patterns induced by sequential finger movement for brain-computer interfaces

**DOI:** 10.3389/fnins.2023.1180471

**Published:** 2023-08-29

**Authors:** Chang Liu, Jia You, Kun Wang, Shanshan Zhang, Yining Huang, Minpeng Xu, Dong Ming

**Affiliations:** ^1^Academy of Medical Engineering and Translational Medicine, Tianjin University, Tianjin, China; ^2^School of Precision Instruments and Optoelectronics Engineering, Tianjin University, Tianjin, China; ^3^International School for Optoelectronic Engineering, Qilu University of Technology (Shandong Academy of Sciences), Jinan, China

**Keywords:** electroencephalography, sequential finger movements, movement related cortical potentials, event-related desynchronization, brain-computer interface

## Abstract

**Objective:**

In recent years, motor imagery-based brain–computer interfaces (MI-BCIs) have developed rapidly due to their great potential in neurological rehabilitation. However, the controllable instruction set limits its application in daily life. To extend the instruction set, we proposed a novel movement-intention encoding paradigm based on sequential finger movement.

**Approach:**

Ten subjects participated in the offline experiment. During the experiment, they were required to press a key sequentially [i.e., Left→Left (LL), Right→Right (RR), Left→Right (LR), and Right→Left (RL)] using the left or right index finger at about 1 s intervals under an auditory prompt of 1 Hz. The movement-related cortical potential (MRCP) and event-related desynchronization (ERD) features were used to investigate the electroencephalography (EEG) variation induced by the sequential finger movement tasks. Twelve subjects participated in an online experiment to verify the feasibility of the proposed paradigm.

**Main results:**

As a result, both the MRCP and ERD features showed the specific temporal–spatial EEG patterns of different sequential finger movement tasks. For the offline experiment, the average classification accuracy of the four tasks was 71.69%, with the highest accuracy of 79.26%. For the online experiment, the average accuracies were 83.33% and 82.71% for LL-versus-RR and LR-versus-RL, respectively.

**Significance:**

This paper demonstrated the feasibility of the proposed sequential finger movement paradigm through offline and online experiments. This study would be helpful for optimizing the encoding method of motor-related EEG information and providing a promising approach to extending the instruction set of the movement intention-based BCIs.

## 1. Introduction

Brain-computer interfaces (BCIs) are the direct communication pathways through which users can interact with the external world utilizing brain activities (Wolpaw et al., 2002; Chaudhary et al., 2016; Coogan and He, 2018; Xu et al., 2021). Over the last few decades, advances in disciplines such as neuroscience and engineering have introduced the BCI as a promising tool for neurorehabilitation and neurophysiology research (Robinson and Vinod, 2016). The BCIs based on decoding motor-related neural activities can be used to drive functional electrical stimulation, intelligent prosthesis, or mechanical exoskeletons, which have important research value for the rehabilitation, replacement, and enhancement of motor functions (Birbaumer et al., 2008; Sebastian-Romagosa et al., 2021; Hernandez-Rojas et al., 2022; Ju et al., 2022; Wang et al., 2022). The motor-related neural activity of the brain can be induced by actual movement or motor imagery (MI). In the existing research, MI-based BCI is the most commonly used research paradigm (Pfurtscheller and Neuper, 2001; Wolpaw et al., 2002).

Currently, electroencephalography (EEG) has become the most widely used monitoring means of BCI due to its non-invasiveness, relatively low cost, and high time resolution (Park et al., 2012; Xu et al., 2018; Meng et al., 2020; He et al., 2022). Movement-related cortical potentials (MRCP) and sensorimotor oscillatory EEG activity (event-related desynchronization/synchronization—ERD/ERS) provide complementary information on the associated motor activity (Savić et al., 2020). Many studies have focused on detecting the pre-motor state of the upper limbs using EEG correlates such as MRCP or ERD (Sburlea et al., 2015). ERD/ERS is a particular time-locked EEG feature for MI tasks, which represents decreases and increases of power in alpha or beta bands. The alpha and beta frequency bands of ERD can be found over the corresponding sensorimotor areas of the brain when people imagine the movements of their limbs (Kosei et al., 2014; Peng et al., 2020; Dai et al., 2022). Jackson et al. found that motor execution shared similar mechanisms with MI. Motor execution can also induce the ERD/ERS features as MI tasks. In addition, movement-related cortical potentials (MRCPs) can be found during the processes of movement. MRCP is one kind of event-related potential (ERP), which is a time and phase-locked feature. Actual movement can evoke more significant MRCP features than MI tasks. Based on the similarity of neural oscillatory patterns of MI and motor execution, we could develop new paradigms and algorithms for movement-intention decoding through actual movement experiments (Jackson et al., 2003; Sochůrková et al., 2006; Katsumata et al., 2009; Sandhya et al., 2014).

Great progress has been made with the MI-BCI technique in recent years, but it still faces many research challenges. The quantity and classification accuracy of controllable instruction sets cannot meet the needs of users to complete most daily life actions (Qiu et al., 2021). So far, most studies have involved only four simple body MI tasks (i.e., left hand, right hand, foot, and tongue movements), with limited alternative paradigms (Townsend et al., 2006; Yang et al., 2009; Xygonakis et al., 2018). To solve the limitations of the instruction sets of MI-BCI, there have been studies on the decoding of complex limb and sequential limb-movement imagination tasks (Zhou et al., 2010; Doud et al., 2011; Yi et al., 2013). Hsu et al. designed a MI normal form of left and right leg steps and proposed a filter bank common space pattern (FBCSP) combined with fuzzy support vector machine type-II method, which achieved 86.25% recognition accuracy on eight subjects (Hsu et al., 2017). However, the existing MI tasks not only increase the operational complexity of the experiment but also make the output time of a single instruction longer, which reduces the decoding efficiency to a certain extent. Therefore, it is necessary to propose a new movement intention encoding paradigm to shorten the time of single instructions and ensure good classification performance at the same time.

As mentioned above, both ERD and MRCP are time-locked features. In addition, they have specific spatial distribution patterns for different limb movements or imagination tasks. Hence, the sequential limb movement paradigms can effectively combine the time-frequency and spatial domains’ movement-related information, which are promising methods to extend the BCI instruction set and enhance the specificity of different task-induced EEG features. Yi et al. designed a sequential compound limb MI paradigm with a mean classification accuracy of 74.14%, while the time of one trial was 6 s (Weibo et al., 2016). Many studies have analyzed the brain activation mechanism of imagining movements of a single limb sequence. It has been found that the effect of learning movement sequences by imagining movements is similar to that of performing the same movement sequences, and the changes in brain activity between the two are consistent (Zhang et al., 2011; Hardwick et al., 2018; Wang et al., 2019; Zhang Q. et al., 2019). Recently, we investigated how data length affected the classification of repeated keystroke tasks with the index finger and found that single-trial EEG induced by the repeated finger movements had good separability (Zhang S, et al., 2019).

Therefore, we proposed a sequential finger movement paradigm for BCI, which was expected to expand the instruction set and shorten the time of single instructions. From the perspective of the time-frequency-spatial domain, this paper analyzed the neural oscillations patterns induced by sequential movement tasks. MRCP and ERD features were extracted effectively based on the common spatial filtering algorithm, such as discriminative canonical pattern matching (DCPM) (Xu et al., 2018; Wang et al., 2020) and filter bank common spatial pattern (FBCSP) (Chin et al., 2008; Ang et al., 2011; Sun et al., 2022). Mutual information analysis was used to select features. Both an offline and an online experiment were carried out to verify the feasibility of the proposed paradigm.

## 2. Materials and methods

### 2.1. Participants

A total of twenty-two subjects (eight males and fourteen females, aged 22–24 years old, all right-handed) participated in the experiments of this study. Among them, ten healthy subjects participated in the offline experiment to analyze the EEG features of sequential finger movement, and twelve subjects participated in the online experiment to evaluate the effectiveness of the proposed paradigm. None of the subjects had a history of neurological disease or movement disorders. The subjects were informed of the experimental procedure and received a letter of acceptance before the study. The study was approved by the ethical committee of Tianjin University.

### 2.2. Design of the experimental paradigm

#### 2.2.1. Offline experiment

During the experiments, the subjects sat quietly in front of a monitor. Their arms were flat on the table and their left and right index fingers were on the keyboard “Z” and “1,” respectively. The display background color was gray to avoid visual stimulation caused by a screen refresh. We tried to make the prompts as small as possible to help subjects focus on the middle of the screen, thus minimizing the eye movement artifacts of subjects during the recording. Before the formal experiments, the subjects were required to practice pressing keys at one-second intervals under an auditory prompt of 1 Hz. The 1 Hz-auditory cues were always present in formal experiments as background sounds.

**Table 1:** Offline and online classification results of two types of keystrokes of 12 subjects (%).

Subject	L→L versus R→R		L→R versus R→L	
	Off-line	On-line	Off-line	On-line
S1	93.30	87.50	93.73	80.00
S2	95.00	77.50	93.89	62.50
S3	95.00	97.50	97.78	87.50
S4	94.97	90.00	96.11	90.00
S5	98.33	70.00	95.56	87.50
S6	94.44	92.50	94.38	95.00
S7	97.22	77.50	95.49	60.00
S8	96.11	80.00	98.33	97.50
S9	97.65	75.00	98.89	87.50
S10	94.97	72.50	98.33	60.00
S11	99.44	90.00	100.00	97.50
S12	96.60	90.00	96.67	87.50
Mean	95.17	83.33	95.24	82.71
Std.	1.72	8.56	1.99	13.48

**Figure 1:**
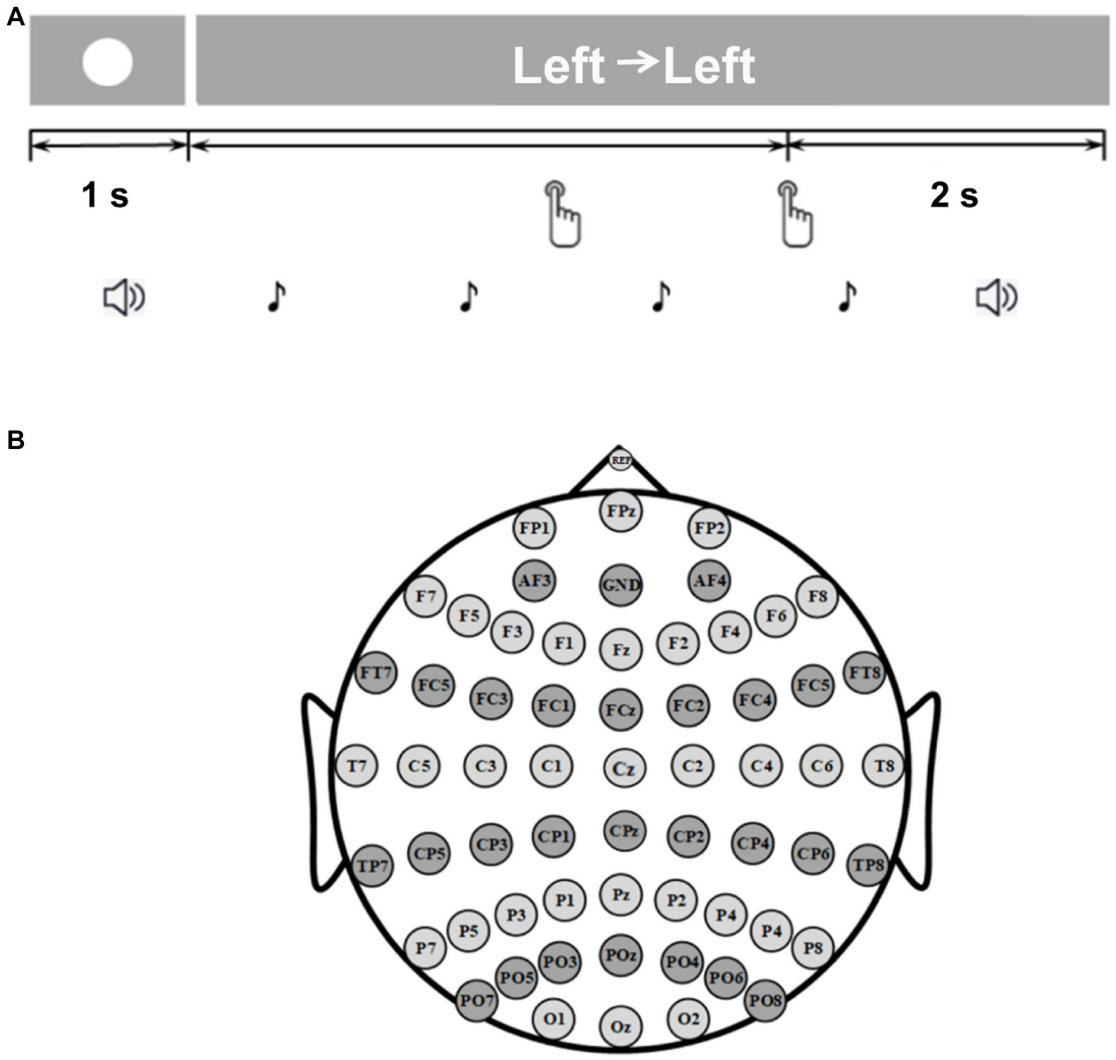
**(A)** The timeline of one trial of the experimental paradigm. **(B)** Locations of the electrodes.

The flow chart of a single experiment trial is shown in Figure 1A. At the beginning of each trial, a white circle appeared in the center of the screen for 1 s to inform the subject that the trial was about to start. After the white circle disappeared, a text prompt appeared. Participants were asked to press the key using the corresponding [i.e., Left→Left (LL), Right→Right (RR), Left→Right (LR), or Right→Left (RL)] index finger in their own time. Subjects were not required to respond immediately to the text prompt. They could decide when to press the button for the first time. For example, if ‘Right → Left’ appeared, the subjects were reminded to press the right-hand key first, and then press the left-hand index finger after an interval of 1 s. There was a 2 s rest period after the subjects completed the keystrokes. During this time, the text prompt remained unchanged. Each participant performed 10 blocks of experiments and each session included 60 trials. Each sequential finger movement task occurred 15 times at random. For each subject, a total of 600 trials (150 trials for each task) were recorded. Trials with wrong key presses or key presses separated by more than 2 s were discarded.

#### 2.2.2. Online experiment

To verify the feasibility of the left and right sequential finger movement paradigm, we performed the online experiment. The online experiment consisted of eight blocks. The procedure of blocks 1 ~ 6 was the same as that of the offline experiment. For each subject, a total of 360 trials (90 trials for each task) were recorded. Two two-class classifiers, i.e., LL-versus-RR and LR-versus-RL, were built using the data from the blocks 1 ~ 6. Session 7 contained 40 trials (20 trials of LL and 20 trials of RR, randomly sorted). The timeline of one trial of session 7 was the same as the offline experiment. During session 7, voice feedback containing the classification result was presented to the subject after the second keystroke of each trial. Session 8 also included 40 trials and had voice feedback following finger movement during each trial. Compared with session 7, session 8 performed 20 trials of LR and 20 trials of RL randomly.

The SVM classifier used in the online experiments was obtained based on offline data training. During the online experiments, each data segment was input to the SVM classifier for classification after pre-processing to extract feature values. The output of the SVM classifier included the predicted category label and its probability score, and we directly used the predicted label as the result output to control the corresponding speech feedback.

### 2.3. Signal recording

In this study, we used a Neuroscan SynAmps2 amplifier to obtain the original EEG signal. The EEG acquisition and amplification device used in this study, manufactured by Compumedics Neuroscan, included a 64-lead EEG cap, a SynAmps2 amplifier, and scan 4.5 software. Sixty Ag/AgCl scalp electrodes were placed according to the international 10–20 system (Figure 1B). The acquisition system referenced the data to the nose, and the prefrontal lobe served as ground. Some skin preparation was required before measurement. If there was dirt or excessive hair on the skin where the electrode was to be placed, the skin should be pre-cleaned or shaved. The sampling rate of EEG signal was 1,000 Hz and the notch filter of 50 Hz was used to eliminate the power frequency interference. We fully checked for bad channels and bad trials (incorrect keystrokes and keystrokes with more than 2 s between them). Bad channels and bad trials were removed if they existed.

### 2.4. Data processing and analysis

Independent component analysis (ICA) is a common blind source separation method in the case of multiple source signals and unknown transmission channel parameters. It functions by observing the signal to estimate the source signal, so as to recover the source signal. Observed signal X (t) = {x1 (t), x2 (t), …, xn (t)} by the source signal S (t) = {s1 (t), s2 (t), …, sn (t)} is obtained by an unknown mixed matrix A, namely, X= AS.ICA is to solve the mixing matrix W when S and A are unknown. At the same time, the estimate Y of the source signal S is separated from the observed signal by W. The prerequisite for ICA is that the number of observed signals is not less than the number of source signals (Song et al., 2022). In this experiment, the influence of eye movement can be seen according to the EMG signal. Therefore, we conducted the ICA process. We used EEGLAB to perform ICA processing on the EEG data and eliminate various artifacts, such as eye movements and blinking. We chose the Runica algorithm for ICA processing. In addition, each subject’s data underwent different bad segment removal operations. To ensure the validity of the ICA processing, we visually inspected each subject’s data and determined the components to be removed based on the EEG waveform and timeline. The number of components removed for each subject was not fixed, but generally ranged from 5 to 10.

In this study, we mainly analyzed MRCPs and ERD features to compare the differences among four different sequence movement-induced patterns of the offline experiment. Since the MRCP potential is a low frequency time-domain waveform signal, we down-sampled the raw EEG data to 16 Hz. Then the data were low-pass filtered at 0–3 Hz using a 4th-order zero-phase Butterworth filter to preserve the low-frequency components of the EEG signal. Common average reference (CAR) was used to improve the signal-to-noise ratio. In this study, we defined the moment of the first keystroke as 0 s and epoched the data from-2 s ~ 3 s for MRCP analysis. The paired t-test was used for statistical analysis of whole subjects between two different sequential finger movement tasks across all time points. To observe the spatial patterns of the four tasks, we calculated the averaged amplitude of all subjects on-150 ms and 850 ms at each channel and plotted the mean topographical distribution across all subjects.

For the ERD analysis, the original signal was down-sampled to 200 Hz and CAR was also applied to it. Then, the signal was bandpass filtered to 4 ~ 30 Hz. The short-time Fourier transform (STFT) of the Hanning window, which has 256 sampling points, was used to calculate the event-related spectral perturbation ERSP between the time range of −1.5 s to 2.5 s for each movement task. We also defined the moment of the first keystroke as 0 and the baseline was the mean of the data ranging from −1.5 s to −1 s. We used the mean ERSP values of all subjects from electrodes C3, Cz, and C4 to compare the time-frequency variation among the four sequential finger movement tasks. Additionally, the averaged alpha band ERSP values of each keystroke in one trial were calculated to analyze the topographical distribution of ERD features. The calculation formula is as follows:

*ERSP* = *ERSP*_original_ − *ERSP*_baseline_

To obtain a higher classification accuracy for single EEG recognition, we needed to utilize some spatial filtering methods to extract both MRCP and ERD features induced by the sequential finger movements. MRCP is a low-frequency waveform feature. Our previous work showed discriminative canonical pattern matching (DCPM) has superiority for MRCP feature extraction. DCPM consists of three major parts: (1) the construction of discriminative spatial patterns (DSPs); (2) the construction of CCA patterns; and (3) pattern matching (Xu et al., 2018). Canonical correlation analysis (CCA) is a multivariate statistical analysis method that uses the correlation between synthetic variable pairs to reflect the overall correlation between two groups of indicators. The CCA algorithm can be used to project the spatially filtered data into a new space and calculate the correlation to reflect the overall correlation of the two groups of indicators (Ma et al., 2022). In addition, other effective feature extraction methods for low-frequency waveform features should also be investigated, such as task-related component analysis (TRCA) (Birbaumer et al., 2008; Nakanishi et al., 2018; Sun et al., 2021), a spatial filtering method for task-dependent component analysis, where the weight coefficients are optimized to maximize the inter-trial covariance of time-locked data. The goal of TRCA is to take task-related constituent parts out from multiple time series that are linearly weighted (Tanaka et al., 2013). For ERD patterns, the filter bank common spatial pattern (FBCSP) was intended to independently select the appropriate frequency bands for feature extracting, which is a popular and effective method (Ang et al., 2012; Chu et al., 2021). The FBCSP method is the optimization of classical spatial filtering in the frequency domain. The effects of different feature selection methods are studied, and the best individual features based on mutual information are used to obtain the selection method with relatively higher classification accuracy (Yong and Wonz, 2019). Hence, we used DCPM and TRCA to extract the MRCP features and used FBCSP to extract the ERD features. Then, we selected the features based on mutual information (Zhang S. et al., 2019).

Before feature extraction, we down-sampled the raw data to 200 Hz first. For each keystroke, we epoched data from 0.5 s ahead of the key stroke and 1.5 s after the key stroke to process, i.e., −0.5 ~ 1.5 s. Different band-pass filters were used for MRCPs and ERD characterization. For MRCPs, we used a band-pass filter (1 to 8 Hz) to filter the data, and then used DCPM and TRCA, respectively, to extract the features. For the ERD features extraction, three crucial characteristic frequency bands, 4 ~ 8 Hz (theta band), 8 ~ 13 Hz (alpha band), and 13 ~ 30 Hz (beta band), were selected for band-pass filtering, and CSP features were extracted, respectively. As the eigenvectors of spatial filters are in descending orders, we selected the first two dimensions for DCPM and the first three dimensions for TRCA and FBCSP. After spatial filtering, we obtained 56 features (16 of DCPM, 4 of TRCA, and 36 of FBCSP) for each trial. In the FBCSP method, the three-dimensional eigenvectors of each CSP filter were selected in three frequency bands for CSP spatial filtering. Therefore, the FBCSP characteristic dimension of the four classifications was 4*3*3 = 36. Furthermore, in order to reduce the characteristic dimension, the mutual information between features and labels was calculated, and pattern recognition was carried out by combining the optimal selection features. The data from the training set was used for feature selection (Aldehim and Wang, 2015). A linear support vector machine (SVM) was used to build the classifier with the help of the famous software package LIBSVM (Yang et al., 2015; Bhatnagar et al., 2016; Dhiman and Priyanka Saini, 2018). We selected the default SVM type and set the penalty factor C to 1. For the offline experiment, we used 10-fold cross-validation to calculate the classification accuracies. For the online experiment, the DCPM, TRCA, and CSP spatial filters were established using EEG data from blocks 1 to 6. We selected 10 features using the mutual information analysis for each subject. Then the online linear SVM classification models were built. All programs were compiled and run on the MATLAB (Matlab used the 2022Rb version of MathWorks) platform. The LL-versus-RR and LR-versus-RL classifiers were applied in the online experiment, respectively. During the online experiment, the EEG data was continuously transmitted to the MATLAB data processing module in real time. The program continuously detected the labels, and then analyzed and processed the data according to the labels. We provided visual feedback during the 2 s break after the second keystroke of each trial, which allowed participants to receive immediate information on their performance. The speech feedback was performed in each trial of the post-processing phase of the data processing program for each trial phase executed, which lasted 100 ms. Finally, the recognition results were fed back to the subjects through voice feedback.

## 3. Results

### 3.1. EEG patterns of sequential finger movement

**Figure 2:**
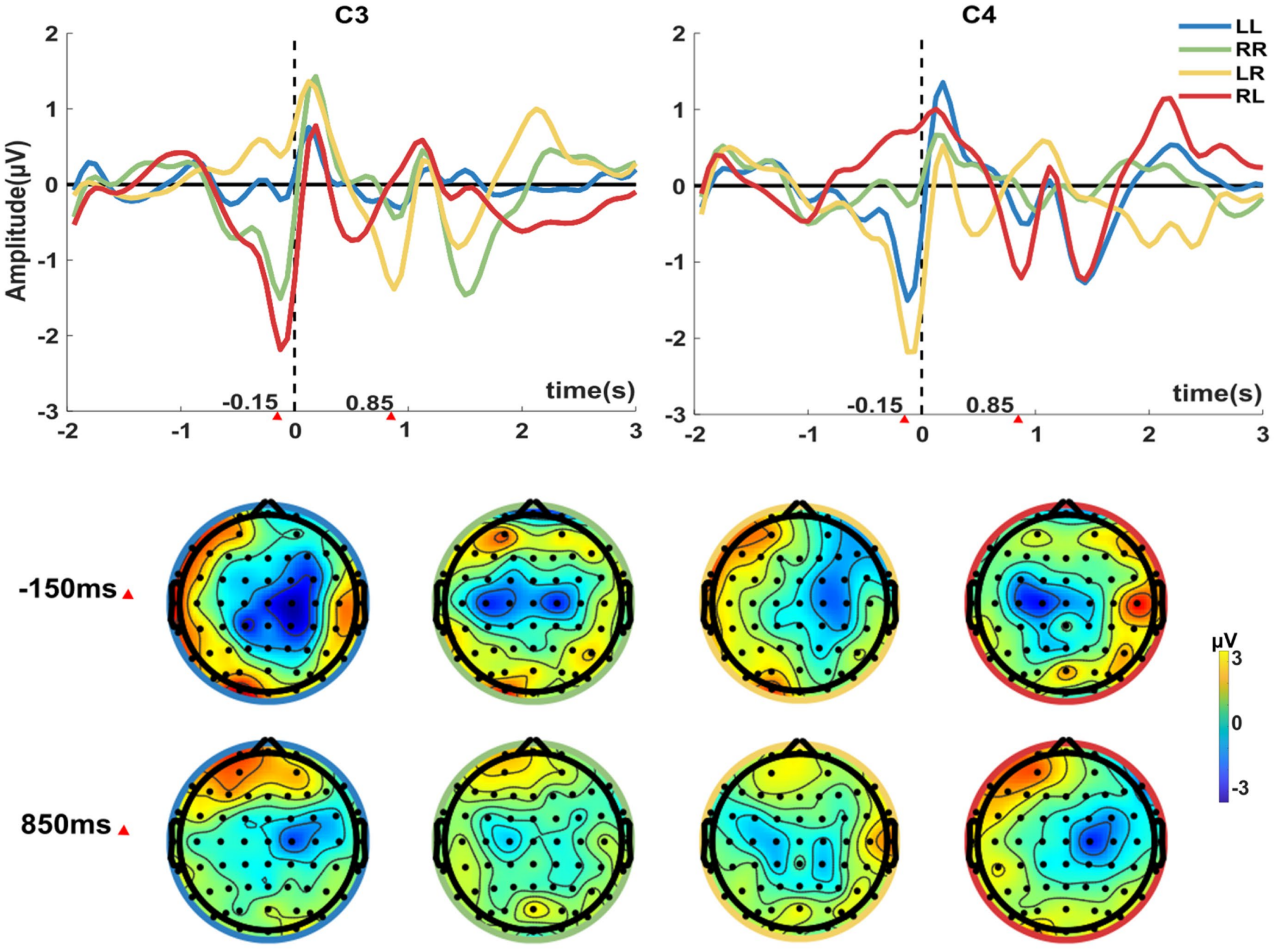
The average MRCPs across all participants of the four sequential finger movements displayed for channels C3 and C4. Time 0 corresponds to the time of the first keystroke. At the bottom of the figure is the spatial distribution of the average MRCP at −150 ms and 850 ms of the 60 channels corresponding to the four-movement tasks. LL (Left→Left), RR (Right→Right), LR (Left→Right), and RL (Right→Left) are used to depict the four tasks, respectively.

We first analyzed the MRCP and ERD patterns induced by sequential finger movement from the offline experiment. The top of Figure 2 shows the average waveforms of MRCPs across all participants of four sequential finger movements at channels C3 and C4. It is obvious that the potentials decreased before the movement onset for both the left and right finger movements, especially for the initial finger. We found that the initial tasks with the left finger, i.e., LL and LR, induced more negative potential on channel C4. On the contrary, right-hand initial finger movement tasks induced more negative potential on C3. This phenomenon also coincided with the contralateral activation of the cortical activity in hand functional areas. For the second sub-action, only the LR and RL tasks showed similar MRCP patterns. In addition, the negative potential peak of the initial keystroke action was lower than that of the non-initial keystroke action.

The bottom of the Figure 2 shows the topography of the average MRCP at −150 ms and 850 ms of the 60 channels. We observed that the channels with the negative waveforms were distributed over the primary motor area and the supplementary motor area. The phenomenon of contralateral dominance could be clearly observed from the topography. MRCP-related negativity induced by the different sub-action tasks (LR and RL) was more pronounced than the repeated sub-action tasks (LL and RR). For the LR and RL tasks, there were completely opposite spatial distributions at −150 ms and 850 ms. Thus, the time-spatial differences of MRCP could be used for classification.

**Figure 3:**
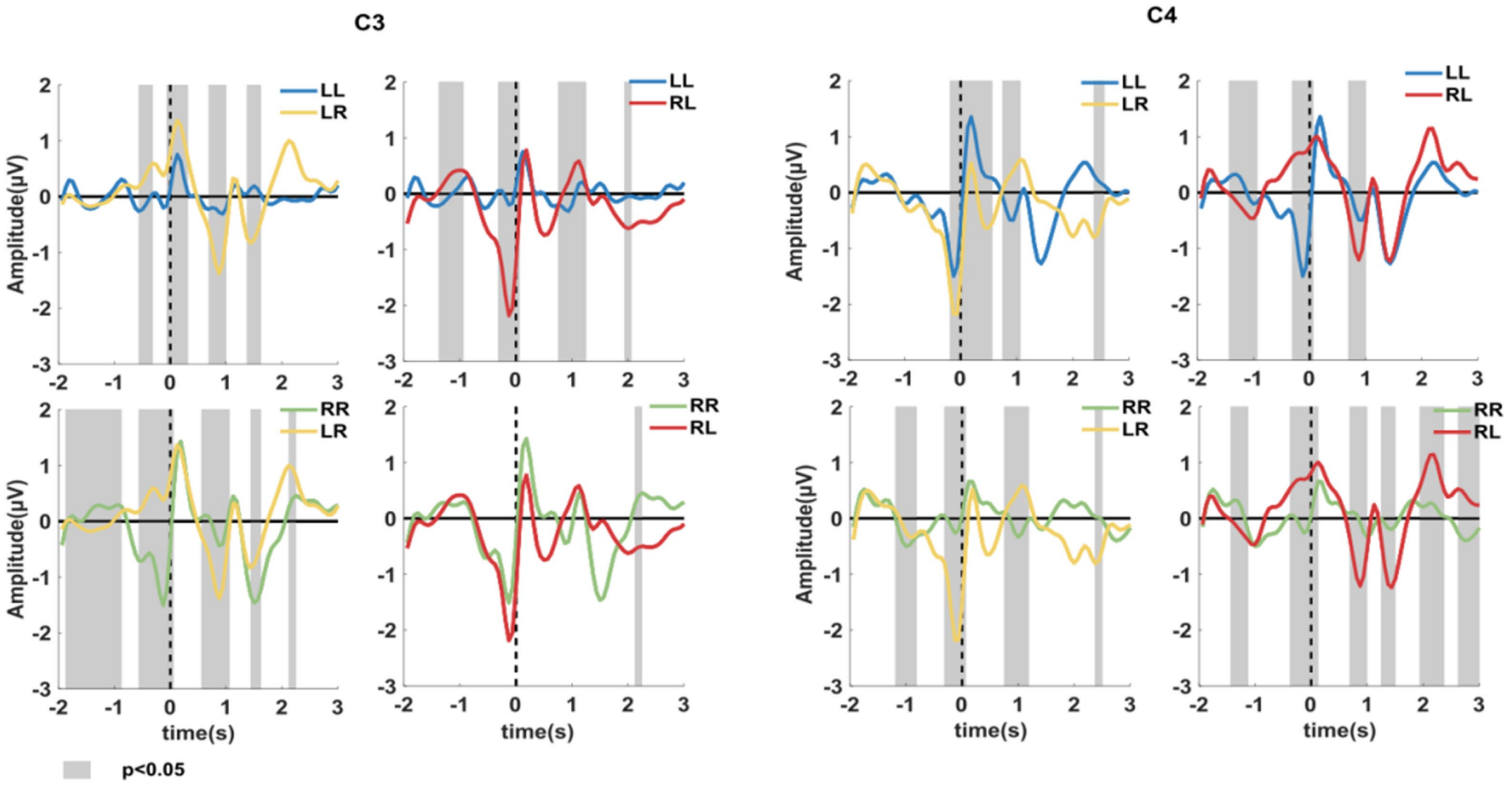
Average MRCP between the different sub-action tasks (LL and RR) and repeated sub-action tasks (LR and RL) at channel C3 and C4. The gray area is the time period with significant difference between the potential amplitudes induced by the two movement tasks (*p* < 0.05, paired *t*-test).

To further investigate the differences between the different sub-action tasks (LR and RL) and the repeated sub-action tasks (LL and RR), the average MRCP potentials between the different sub-actions and the repeated sub-action tasks were, respectively, drawn and analyzed using the paired t-test, as shown in Figure 3. The grey area is the time period with significant difference between the two types of sequential finger movements-induced potential amplitudes. As can be seen from the figure, when the initial sub-action was left finger movement, Bereitschaftspotential (BP) induced by LR and RL tasks were significantly more obvious than those induced by LL task on C3 and C4 channels. Similarly, it could be seen that BP amplitudes before LR and RL tasks were significantly larger than those before RR tasks except RR-versus-RL at C3. These results showed that, compared with simple sequential movement, complex sequential movement might induce stronger MRCP patterns.

**Figure 4:**
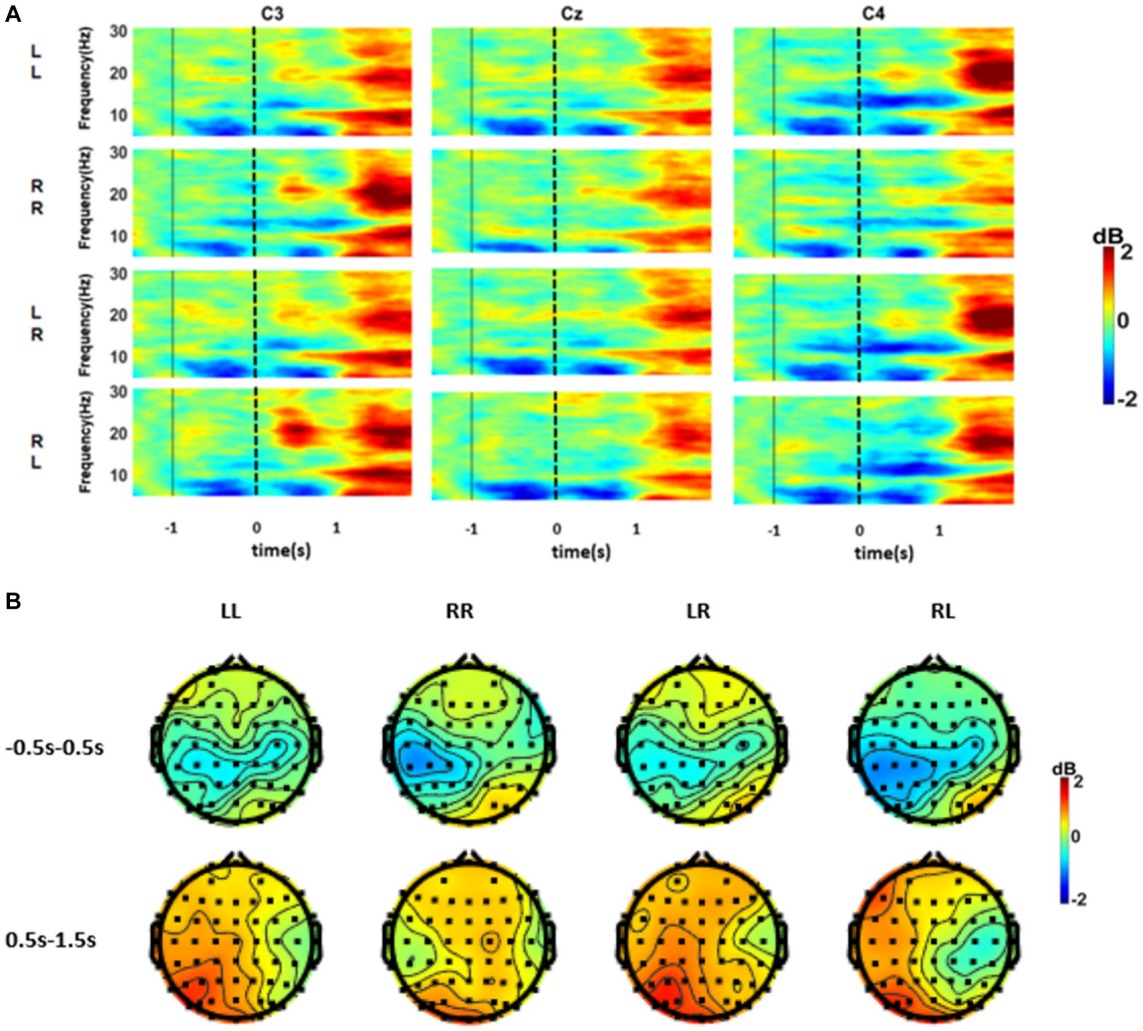
**(A)** Average time-frequency maps of the four finger movement tasks at channels C3, Cz, and C4. Time 0 corresponds to the time of the first keystroke. Blue is ERD, and red is ERS. **(B)** The average 8–13 Hz ERSP topography of the four movement tasks. Among them, −0.5 ~ 0.5 s and 0.5 ~ 1.5 s correspond to the first and second sub-actions, respectively.

Figure 4A shows the average time-frequency graph of four types of index finger sequence movements of 10 subjects in the offline experiment at key channels C3, Cz, and C4. In the figure, it can be observed that all the four movement tasks could induce obvious ERD phenomena in the theta, alpha, and beta bands. Notably, the intensity of ERD patterns varied over time. The ERD phenomenon in theta and alpha bands mainly occurred within 1 s before keystroke, which was of high intensity and involved a wide range. However, there was no significant difference among the three channels. For the repeated sub-action tasks (LR and RL), we found distinct contralateral hemispheric dominance, which was not obvious for the different sub-action tasks (LR and RL). Figure 4B shows the mean alpha band EEG power topography among four different finger movement tasks. It can be seen from the brain topographic map that the LL and RR tasks could activate the motor functional areas of both hands, which showed obvious contralateral dominance. In addition, the ERD intensity induced by the initial sub-action was greater than that of the second sub-action.

### 3.2. Classification performance of offline experiment

**Figure 5:**
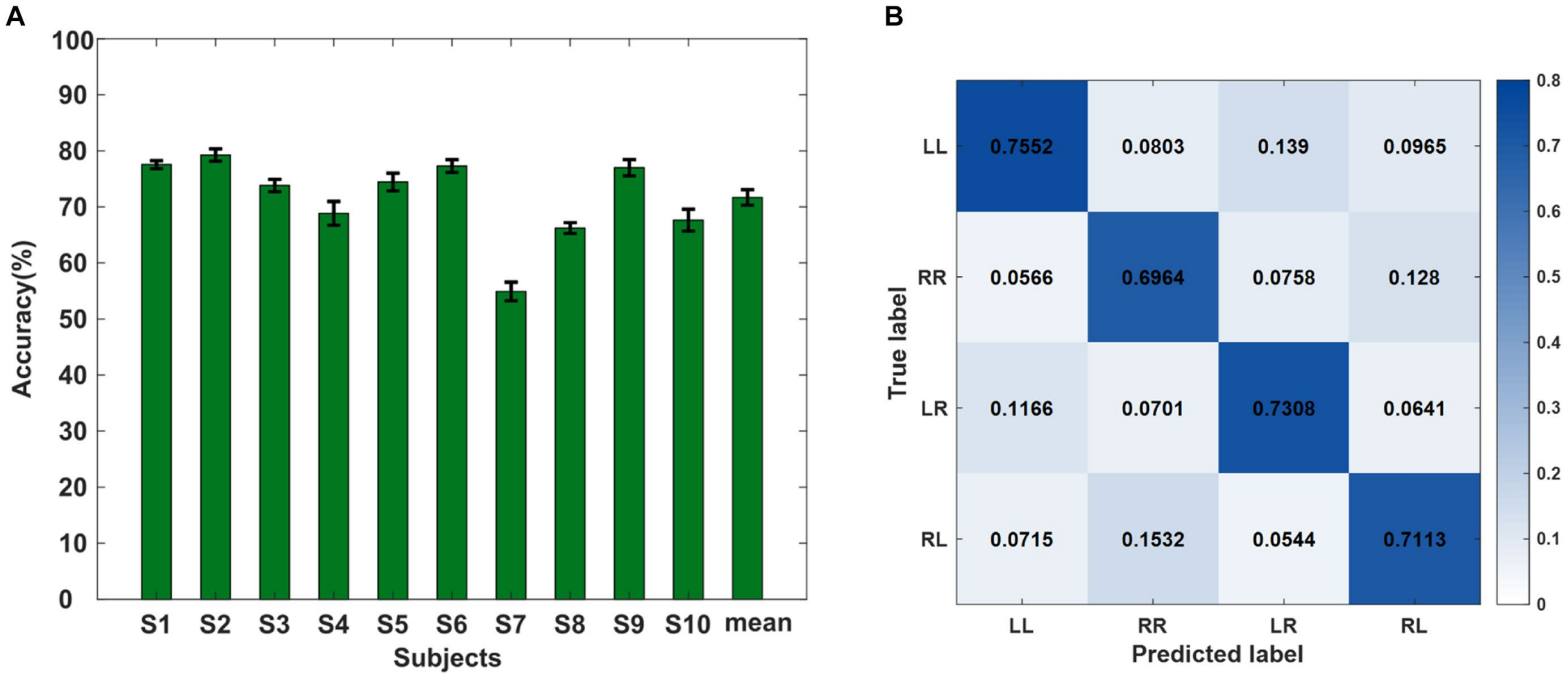
**(A)** Ten-fold classification accuracies (%) for all subjects of the offline experiment. **(B)** Confusion matrix of the average recognition accuracies of the four kinds of sequence finger movements of all subjects. Each row represents the true label and each column represents the predicted label.

For the classification of the data in the offline experiment, the optimal filter dimension and characteristic dimension were selected. Figure 5A shows the classification accuracy results of the four sequential finger movement tasks. The mean classification accuracy of the four classes was 71.69%, which was much higher than the random level of 25%. The highest accuracy was 79.26% and the lowest accuracy was 54.91%.

We also calculated the confusion matrix under the optimal feature dimension of the four categories, as shown in Figure 5B. Each row represents the true label and each column represents the output result. The figure shows the proportion of each type of task divided into four different results by the classifier. It shows that the percentage of classification errors varied from task to task. The distribution of the four action task features in the classifier was not irregular but a regular distribution in a certain projection direction. At the same time, it can be seen that the four types of sequential finger movement tasks had different classification difficulties. The two types of tasks with two different sub-actions were easier to distinguish than the sequences with the same sub-actions. When subjects performed RL or LR tasks, the false recognition of RR had a higher occurrence rate than the LL task.

### 3.3. Classification performance of online experiment

**Figure 6:**
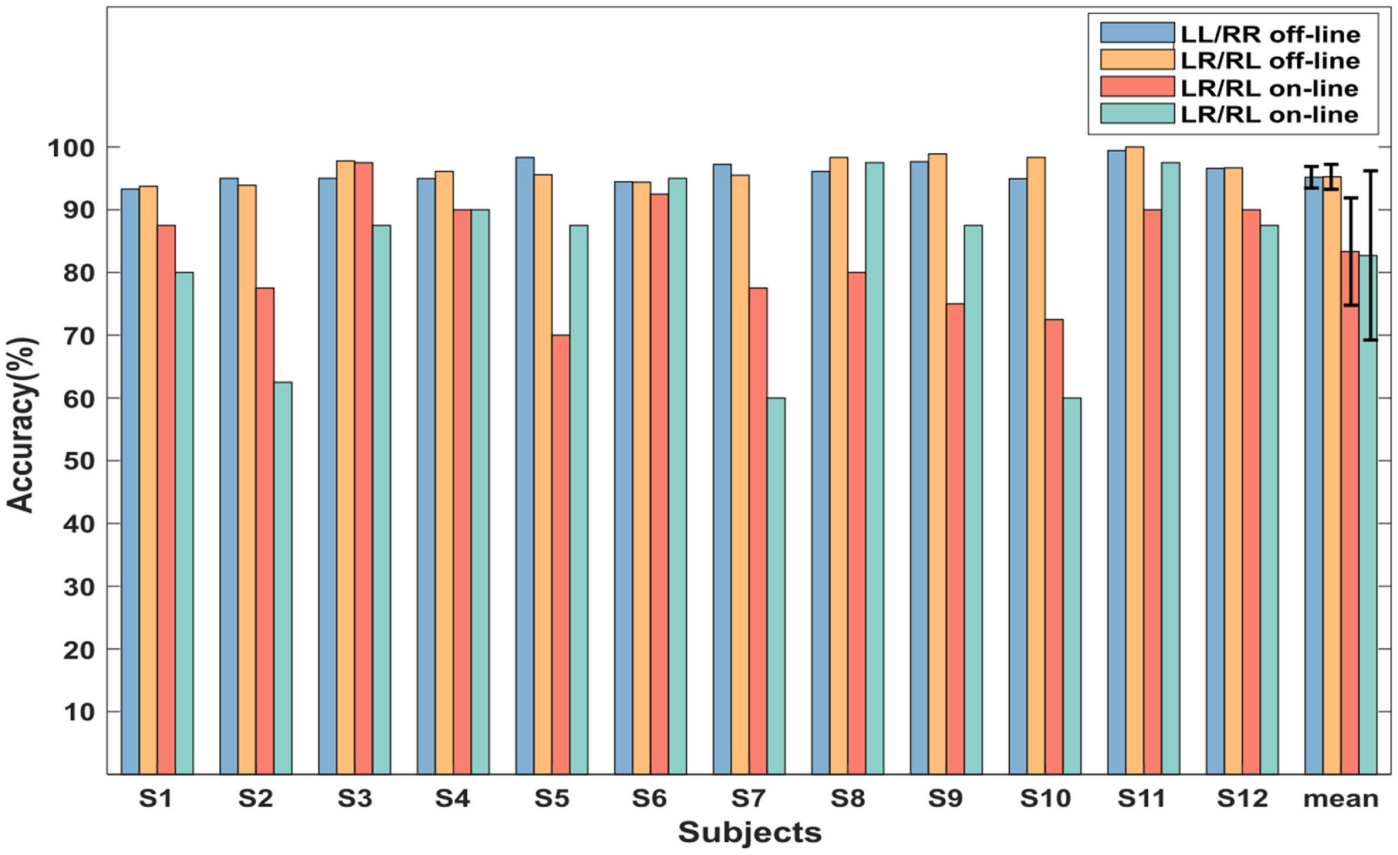
The offline and online experimental classification accuracies (%) of all subjects.

The online experimental results of 12 subjects are shown in Figure 6 and Table 1. The online recognition accuracies were 83.33 and 82.71% for LL-versus-RR and LR-versus-RL, respectively. The classification results of S4, S6, and S11 in the two types of online experiments all reached more than 90%, which proved the feasibility of the sequential finger movement paradigm proposed in this study. However, the classification accuracies of S2, S5, S7, S9, and S10 decreased significantly compared with the offline model. This was caused by the overfitting of the model. Due to the non-stationarity of MI-EEG signals, there may be significant differences in EEG features between the training and testing datasets. Therefore, a classification model constructed through the training set may not adapt well to the testing set.

## 4. Discussion

This paper explored expanding the instruction set for movement intention-related BCIs. This paper showed that the sequential movement of the left and right fingers could induce the distinguishable MRCP and ERD features containing time-frequency-spatial movement-related information. In our previous study, we combined the MRCP and ERD to decode the pre-movement EEG patterns of left and right finger movement and obtained a satisfactory performance (Wang et al., 2020). However, that study could only be used for binary classification. In this study, the cortical activation of the left and right index finger keystrokes were predominated contralateral, which was consistent with the results of some other studies (Zang et al., 2003; Francesco et al., 2005; Bian et al., 2022). In addition, we also found the EEG spatial patterns were rhythmically changed with the rhythmically sequential tasks (Figures 2, 4). Hence, sequential finger movement adds information encoding in the time domain, which is meaningful to extend the instruction of movement intentions.

In Figure 2, we can see the negative potential peak of the initial keystroke action was obviously lower than that of the non-initial keystroke action, especially for the repeated sub-action tasks (LL and RR). The reasons for this phenomenon might come from two aspects. On the one hand, we epoched all trials using the label of the first keystroke. Although we set a 1 Hz background sound cue during the experiment, the time between the second keystroke and the first keystroke had a certain error compared with 1 s. Hence, for the non-initial keystroke, the negative potential may not be so pronounced after calculating the mean wave because the data were not perfectly aligned. On the other hand, compared with the different sub-action tasks (LR and RL), the subjects were more familiar with the non-initial keystroke action due to it being the same as the initial action for the repeated sub-action tasks, which might have resulted in less activation of the brain cortex. Jancke et al. showed that repeated practice of an action has an effect on motor cortex activation, and familiar action-induced ERD features were reduced (Jancke et al., 2006). This phenomenon may be similar to the repetitive inhibitory effect of steady-state visual-evoked potentials (Xu et al., 2022).

In Figure 4, the onset time of ERD in the alpha band is earlier than that in the beta band. Some studies indicated that the amplitude of alpha-band oscillations significantly decreased over the motor regions that began in the motor preparation stage, which implied that the alpha rhythm was more relevant to motor planning/programming (Kajihara et al., 2015). The rhythmically sequential movement we proposed in this paper is the more sophisticated motor control. Hence, the cortex associated with motor planning and related advanced cognitive activities would be active before movement.

**Figure 7:**
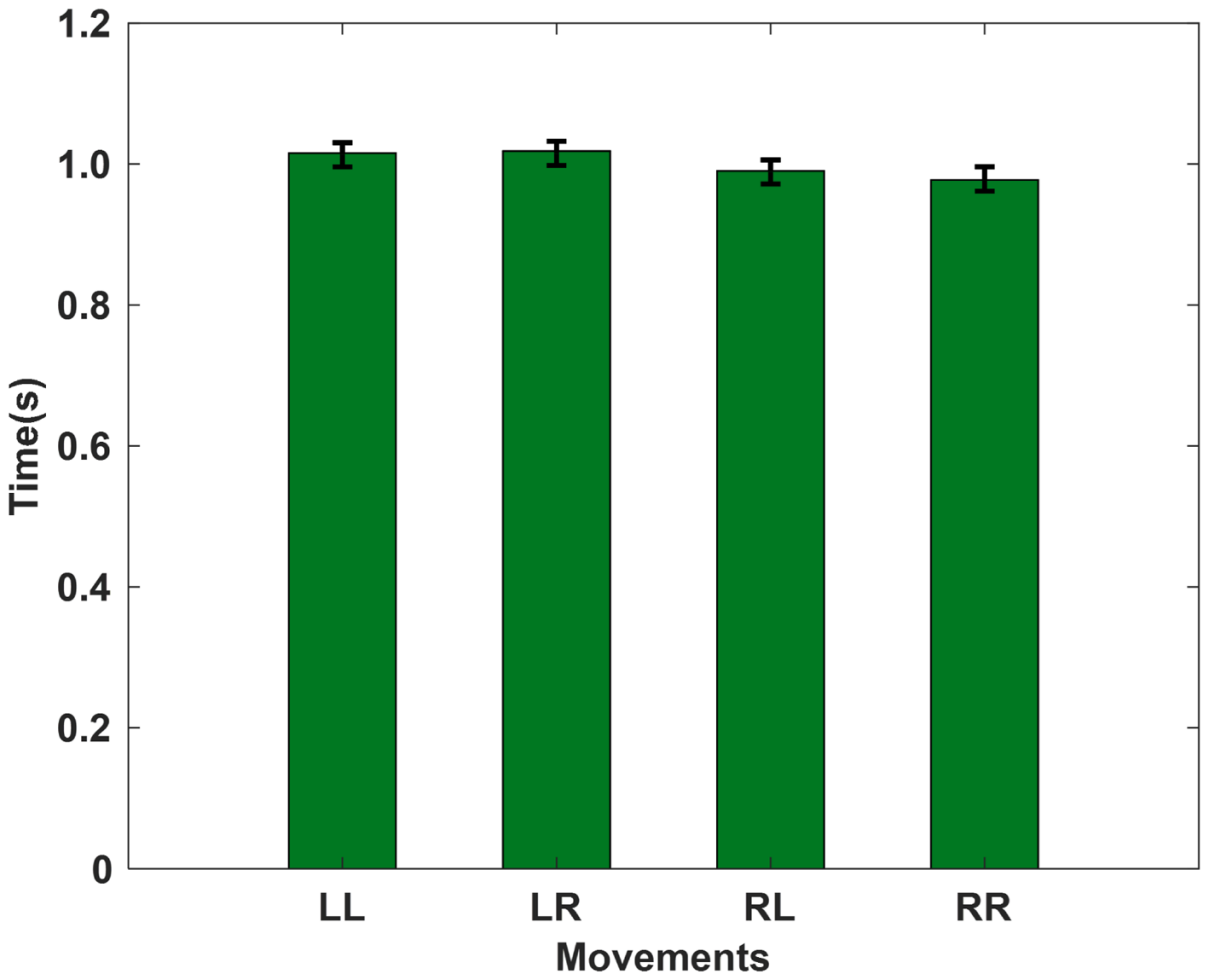
The average time difference between subjects’ first and second keystrokes for each sequential action task.

We conducted behavioral analysis. The time difference between the two keystrokes was analyzed statistically. The results showed that there was no significant difference between the two keystrokes under different tasks. The results are shown in Figure 7.

The classification results showed that the LL-versus-RR and LR-versus-RL are the two classification models with better performance. This is mainly due to the difference between the preceding sub-actions and the following sub-actions of the above two models. Secondly, we found that the classification models with the different initial sub-action (LL-versus-RL or RR-versus-LR) had better performance than that with different non-initial sub-actions (LL-versus-LR or RR-versus-RL). This indicated that the initial sub-action can provide more classification information than the non-initial sub-action in the sequential movement paradigm. In this paper, although the mean four-classification accuracy of the offline experiment has shown divisibility, it still does not meet the needs of external device control for everyday BCIs. Therefore, the online experiment mainly focused on the two best-performing binary models. The existing work is still in the preliminary stage. In the future, the classification algorithm should be further optimized and improved to realize the high classification accuracy meeting everyday BCI use with a large instruction set. Considering the similarity in EEG patterns between MI and real movement, how to transfer the sequential movement paradigm to sequential MI is also a problem worth exploring in the future.

The difference between the results of the online and offline experiments may be due to the non-linear and non-stationary characteristics of the EEG signals. The offline results were obtained by 10-fold cross validation calculation, and the training data were close to the test data so that it had a high similarity. In the online experiment, the training data used to build the classification model were completely separated from the test data. Consequently, the online experimental classification effect of subjects whose EEG signals changed greatly over time was poor.

The experimental paradigm we are using now is the motor execution of the subject performing a real action. Subsequently, we will use transfer learning to make subjects realize brain-computer interactive control through motor imagination. For the subsequent application of motor imagination, it can be used to help patients with rehabilitation, assistance, etc.

However, when we switch from motor execution to motor imagination, the EEG signal might be weakened. In addition, there could be some problems such as inaccurate time labels. Wu et al. studied the problems of applying transfer learning to brain-computer interfaces and how to solve them (Wu et al., 2022).

## 5. Conclusion

This paper demonstrated the feasibility of the proposed sequential finger movement paradigm, which had a satisfactory performance on recognition. The spatial distributions of both MRCPs and ERD were varied regularly with the different finger movements. In general, this study proposed a promising encoding method of movement intention to improve the discriminated information dimension of EEG patterns, which might provide a new idea and theoretical basis for effectively expanding the command set of movement intention-related BCIs.

## Data availability statement

The raw data supporting the conclusions of this article will be made available by the authors, without undue reservation.

## Ethics statement

The studies involving human participants were reviewed and approved by the Research Ethics Committee of Tianjin University. The patients/participants provided their written informed consent to participate in this study.

## Author contributions

CL, JY, KW, MX, SZ, YH, and DM conceived the study. CL, JY, and KW designed and conducted the experiments. CL and SZ performed data analyses. CL and KW wrote and edited the initial draft. MX, KW, and DM performed proofreading and the finalizing of the manuscript. All authors contributed to the article and approved the submitted version.

## Funding

This work was supported by the STI 2030—Major Projects 2022ZD0208900, National Natural Science Foundation of China (No. 62206198, 62122059, and 81925020), and Introduce Innovative Teams of 2021 “New High School 20 Items” Project (2021GXRC071).

## Conflict of interest

The authors declare that the research was conducted in the absence of any commercial or financial relationships that could be construed as a potential conflict of interest.

## Publisher’s note

All claims expressed in this article are solely those of the authors and do not necessarily represent those of their affiliated organizations, or those of the publisher, the editors and the reviewers. Any product that may be evaluated in this article, or claim that may be made by its manufacturer, is not guaranteed or endorsed by the publisher.
